# Does the clamping method in local and systemic TXA applications in total knee arthroplasty change the game?: A retrospective comparative cohort study

**DOI:** 10.1097/MD.0000000000030823

**Published:** 2022-09-23

**Authors:** Kaya Turan, Osman Görkem Muratoğlu, Tuğrul Ergün, Haluk Çabuk, Ramazan Erden Ertürer

**Affiliations:** a Department of Orthopedics and Traumatology, Medicine Faculty of Istinye University, İstanbul, Turkey; b Department of Orthopedics and Traumatology, Istinye University Training and Research Hospital, İstanbul, Turkey.

**Keywords:** arthroplasty, blood loss, drain clamping, knee, tranexamic acid

## Abstract

Many different methods and drain clamping periods have been described in systemic and local tranexamic acid (TXA) applications, and the superiority of the methods to each other has not been clearly demonstrated. The method of local infusion in combined TXA applications may not alter the Hb drop or total or hidden blood loss. We aim to compare two different combined TXA application methods. We retrospectively analyzed 182 patients who underwent total knee arthroplasty between 2018 and 2021. Patients over 40 years of age who underwent TKA for degenerative knee arthritis were included in the study. Unicondylar, revision, or bilateral arthroplasties and patients with the cardiovascular or cerebrovascular disease were excluded from the study. All patients in the study received 1 g TXA intravenously half an hour before the incision. For the first group, 1 g TXA was given intra-articularly at the drain site after closure, and the clamp was kept closed for 1 hour. In the second group, the drain was clamped for an additional 6 hours, and a 1 g intravenous dose was administered at the 5th hour postoperatively. No local applications were used in the control group. Total, hidden, and visible blood loss (total blood loss, hidden blood loss, visible blood loss), postoperative decreases in hemoglobin and hematocrit level (ΔHgb, ΔHtc), blood transfusion rates, and hospital stay durations were evaluated. There were 72 patients in the first group, 52 in the second, and 58 in control. A total of 37 patients received one or more blood transfusions postoperatively, and there was no statistical difference in the need for blood transfusions between the groups (*P* = .255). Although a statistically significant difference (*P* = .001) in total blood loss, hidden blood loss, visible blood loss and ΔHgb values was observed between the groups, the difference between the first and second groups was insignificant (*P* = .512). The duration of hospital stay was observed to be less in the first and second groups (*P* = .024). Local and systemic TXA applications were observed to be more effective than only systemic applications in reducing blood loss after total knee arthroplasty, regardless of the local method.

## 1. Introduction

Total knee arthroplasty (TKA) is the last-line treatment for patients suffering from osteoarthritis-related pain. With the development of arthroplasty techniques and prosthesis designs, better functional results can be obtained in elderly patients and prosthesis survival is also prolonged.^[1]^ One of the major drawbacks for patients undergoing TKA is the perioperative bleeding and related complications due to blood loss; the estimated amount of bleeding after TKA varies between 800 and 1800 mL.^[2]^ Allogeneic blood transfusions bring many risks and problems, including infections transmitted through blood, immunological reactions, prolonged hospitalization, blood product supply chain difficulties, and increased costs.^[3]^

Antifibrinolytic drugs reduce blood loss and reduce the risks associated with blood transfusions. Tranexamic acid (TXA) is an antifibrinolytic drug used in many surgical procedures to reduce bleeding and total blood loss (TBL). A synthetic derivative of lysine, TXA inhibits the conversion of plasminogen to plasmin by blocking the lysine-binding sites, while plasminogen binds to fibrin, thus inhibiting fibrinolysis. Clot stabilization results from decreasing the proteolytic effect on fibrin monomers and fibrinogen.^[4]^ TXA is administered systemically, orally, or intravenously. The topical application involves a direct application to bleeding wound surfaces, reducing the side effects of systemic application.^[2]^ A recent study showed that intravenous TXA administrations are more effective in reducing postoperative hemoglobin (Hgb) decrease and the amount of bleeding from the drain.^[5]^ In contrast, intra-articular TXA applications have been shown to be more effective in reducing both Hgb decrease and joint swelling after TKA.^[6]^ In a randomized controlled study on combined applications in which three methods—one using intravenous, one using intra-articular, and one using both—were compared, the most effective method to guard against Hgb decrease was intravenous and intra-articular administration.

TXA has minimal side effects compared to other antifibrinolytic agents.^[4]^ An increase in deep vein thrombosis, pulmonary embolism, and thromboembolic events associated with TXA use have not been demonstrated.^[7,8]^

Previous cardiovascular and orthopedic surgery studies have shown TXA to reduce blood loss and postoperative blood transfusion rates without major complications^[9–11]^; many meta-analyses have confirmed these results.^[9,12,13]^ However, few studies have compared systemic local or combined TXA application methods employed in TKA.^[5,14–16]^ Although there is no clear consensus on the ideal dosing regimen, including dose, timing, and route of administration, it is evident that it reduces the need for blood transfusions, whether local or systemic.^[17]^ We hypothesized that the method of local infusion in combined TXA applications does not alter the Hb drop or total or hidden blood loss. In this context, our study aimed to compare two different combined TXA application methods and demonstrate their effectiveness in maintaining Hgb levels after TKA.

## 2. Materials and Methods

**Figure 1.**
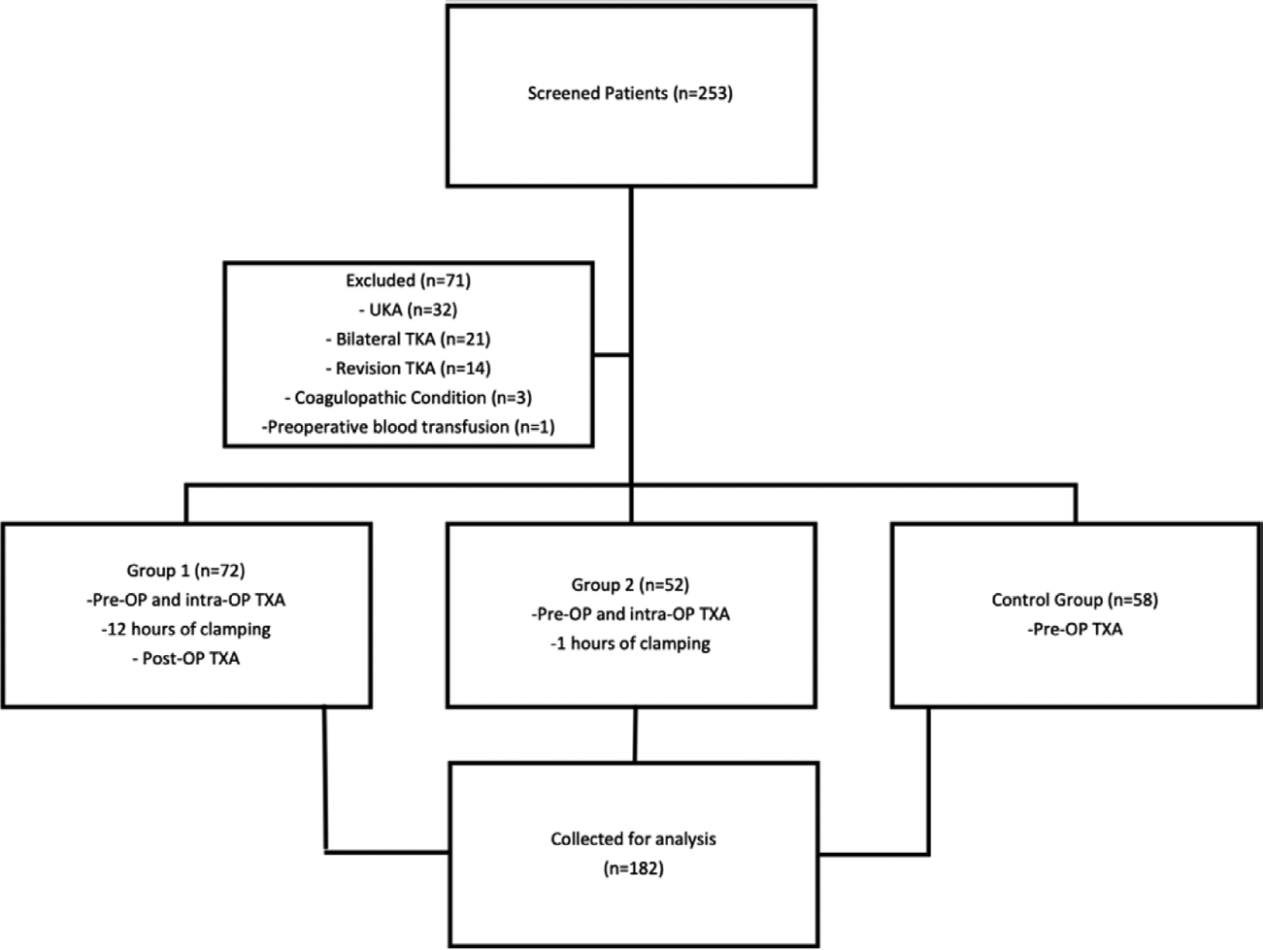
Flowchart of the study planning. Intra-OP = Intraoperative, OP = operative, Pre-OP = Preoperative, Post-OP = Postoperative, TKA = total knee arthroplasty, TXA = tranexamic acid, UKA = Unicompartmental arthroplasty.

In our study, 186 patients who underwent unilateral bicompartmental cemented TKA in a tertiary orthopedic surgery department between 2017 and 2020 were evaluated retrospectively with the approval of the local university clinical research ethics committee. Patients over 40 years of age who underwent TKA for degenerative knee arthritis were included in the study. Patients who underwent unicondylar arthroplasty, revision arthroplasty, bilateral surgery, have cardiovascular problems (myocardial infarction, atrial fibrillation, angina, etc.), patients with a history of cerebrovascular disease, and patients with thromboembolic disorders were excluded from the study. The patients were divided into three groups according to the TXA method applied (Fig. 1). The demographic characteristics of the groups were recorded.

All cases were performed under spinal anesthesia, and patients who underwent general anesthesia were not included in the study. All surgical procedures were performed by three different orthopedic and traumatology specialists with a high annual arthroplasty case volume. As a standard, medial parapatellar arthrotomy was performed with a tourniquet in the surgical approach. An intramedullary guide for the femur and an extramedullary guide for the tibia was used to resect the bone. The patellar surface replacement was not performed, cemented posterior stabilizing prostheses were used in all cases regardless of the severity of deformity and arthrosis (Zimmer^®^ NexGen^®^ LPS, Zimmer Biomet Vanguard^®^, Smith&Nephew Genesis II^®^).

The first group included patients who received 1 g of intravenous application half an hour before the incision and 1 g of intra-articular application through the drain after closure. The drain clamp was opened after being closed for 1 hour. The second group included patients who received 1 g intravenous TXA half an hour before the incision; after closure, the drain was clamped in these patients by applying 1 g intra-articular TXA through the drain, then opened 1 hour after 1 g intravenous TXA was applied again at the 5th hour postoperatively. The third group included patients evaluated as controls; these patients received systemic 1 g intravenous TXA preoperatively. The tourniquets of the patients in the first and control groups were not lowered until the end of the closure.

For the patients in the second group, the tourniquet was tightened before the component placement and lowered after the closure, without using a tourniquet until the implant placement. A hemovac drain was placed in the suprapatellar pouch. After the operation, thromboembolism prophylaxis was applied with enoxaparin and anti-embolism stockings appropriate to body weight, and mobilization was initiated on the first day with joint range of motion and isometric exercises. The preoperative Hgb values of the patients and the lowest postoperative Hgb values were recorded. Hgb and hematocrit were measured preoperatively and at the 12th, 24th, and 48th hours after surgery. According to the formula by Xu et al,^[18]^ TBL, hidden blood loss, and visible blood loss were calculated. Postoperative complications were recorded, such as frequency and number of transfusions, infection, skin problems, deep vein thrombosis, and pulmonary embolism. Transfusion was applied to patients with a Hgb level below 8.0 g/dL or patients with accompanying anemic symptoms and a Hgb value below 10.0 g/dL.

A statistical power analysis was performed for sample size estimation by GPower (ver 3.1) based on our pilot data comparing ΔHgb with the means and SD (±1.06) within each group. The effect size in this study was 0.547. With an alpha = 0.05 and power = 0.85, the projected sample size needed with this effect size is approximately N = 49 for between-group comparison. Quantitative data were compared among the three groups by one-way analysis of variance and Tukey-HSD post hoc analysis. SPSS is version 22.0 for MacOs (IBM Corporation, Armonk, NY) was used for all analyses, with a *P* value for statistical significance <.05. The study was approved by the university’s clinical research ethics committee.

## 3. Results

Our study evaluated the data of 182 patients, 134 women and 48 men. There were 72 patients in the first group, 52 in the second group, and 58 in the control group. No statistical differences were observed in the patient’s preoperative Hgb and hematocrit levels. ΔHgb values were found to be 2.7 ± 0.9, 3.7 ± 1 and 4.5 ± 1.2 in the first, second, and control groups. The decrease in Hgb, TBL, and hidden blood loss was significantly less in the first two groups compared to the control group (*P* < .05), with no significant difference between the first and the second groups (*P* = .512). Table 1 presents the mean Hgb decreases between the groups.

**Table 1:** Clinical and surgery-related data of the groups.

	Group 1 (n = 72)	Group 2 (n = 52)	Control group (n = 58)	*P* value ^*^
Age ± SD (yr)	62.89 ± 7.60	61.42 ± 8.72	65.11 ± 8.80	.312
Gender				
Male	19	13	16	.955
Female	53	39	42	
Side L:Left, R: Right, Bil : Bilateral (L/R/Bil)	27/32/13	20/17/15	28/14/16	.064
Height ± SD (m)	1.54 ± 0.12	1.53 ± 0.09	1.58 ± 0.31	.114
Weight ± SD (kg)	65.49 ± 10.50	62.76 ± 11.95	63.64 ± 9.50	.189
BMI ± SD (kg/m^2^)	26.06 ± 3.78	25.22 ± 3.94	26.54 ± 4.41	.228
Operative time ± SD (min)	95.12 ± 37.16	91.76 ± 25.83	90.35 ± 40.22	.330
Preoperative mean				
Hgb (g/dL)	12.62 ± 1.88	12.61 ± 1.35	12.80 ± 1.40	.764
Hct	39.05 ± 5.01	38.11 ± 3.60	38.43 ± 3.73	.451
Postoperative mean				
Hgb (g/dL)	10.53 ± 1.28	11.10 ± 1.49	10.47 ± 1.24	.027^*^
Hct	32.45 ± 3.90	33.53 ± 4.19	31.47 ± 3.52	.022^*^
Δ Hgb	2.08 ± 1.34	1.50 ± 1.50	2.32 ± 0.98	.001^*^
Δ Hct	6.60 ± 3.96	4.57 ± 2.73	6.96 ± 2.81	.001^*^
HBL (mL)	275.75 ± 151.14	257.13 ± 61.14	339.50 ± 101.84	.001^*^
VBL (mL)	389.60 ± 186.2	331.52 ± 96.7	396.80 ± 114.8	.001^*^
TBL (mL)	665.66 ± 121.76	588.65 ± 100.18	736.35 ± 272.81	.001^*^
Length of stay (d)	6.01 ± 3.37	4.77 ± 1.84	5.48 ± 1.85	.024^*^

Bil. = Bilateral, BMI = body mass index, HBL = hidden blood loss, Hct = hematocrit, Hgb = hemoglobin, L = Left, R =Right, SD = standard deviation, TBL = total blood loss, VBL = visible blood loss.

* One-way ANOVA analysis.

Though it was observed that 5 patients in the first group, 4 patients in the second group, and 10 patients in the control group needed a transfusion, the difference among the groups was not statistically significant (*P* > .05) (Table 2). The duration of hospital stay was found to be shorter in the second group than in the third group (*P* = .027).

**Table 2:** Effect of tranexamic acid on postoperative blood transfusion.

Group	No. of patients having transfusion	No. of units transfused	*P* value
Group 1 (n = 72)	19	30	.594
Group 2 (n = 52)	8	16	
Control group (n = 58)	5	8	

No thromboembolic events or infections developed in patients in the early postoperative period. In two patients in the second group and three in the control group, the hospital stay was prolonged due to prolonged wound leakage. However, no additional problems were encountered during follow-up.

## 4. Discussion

The most important finding of our study is that TXA applications reduce postoperative Hgb decreases in TKA. Another important conclusion of our study is that combining the local and systemic applications is more effective in reducing blood loss. It was also determined that in local applications, keeping the drain clamped for a longer time or administering an additional dose of intravenous TXA has no beneficial effect over a single local application. Although there are many prospective randomized studies in the literature comparing local and systemic applications, as far as we know, there is no study in which the clamping time in local and systemic applications is different and with an additional systemic infusion compared with the control group.

Fibrinolysis is an essential physiological process that maintains hemostatic balance and prevents thrombosis.^[15]^ This process is simultaneous with the post-injury coagulation cascade and maintains hemostasis by preventing clot growth in damaged areas and clearing mature fibrin. Pathologically, excessive activation of the fibrinolytic process is defined as hyperfibrinolysis, which causes insufficient clot formation.^[19]^ It has been reported that hyperfibrinolysis occurs within the first few hours after injury and causes an increase in blood loss.^[20]^ Current evidence indicates that surgical trauma caused by TKA significantly activates the fibrinolytic system.^[21]^ It has been determined that hyperfibrinolysis peaked with D-Dimer levels in the first 24 hours after surgery.^[22]^

TXA is an antifibrinolytic drug. When administered intravenously, it widely affects the intracellular and extracellular compartments,^[23]^ penetrating the synovial fluid rapidly and reaching its serum concentration within the joint.^[24]^ It’s biological half-life in joint fluid is approximately 3 hours.^[24]^ After intravenous administration of 10 mg/kg, TXA is excreted from the body by glomerular filtration at a rate of 30% in the first hour, 55% in the third hour, and 90% in the 24th hour.^[23]^ The advantage of topical TXA is that it is applied directly to the bleeding site, but after surgical hemostasis is achieved, lowering the tourniquet results in significantly increased local fibrinolysis^[25]^; inhibiting this not only helps to prevent fibrin clot dissolution but also provides microvascular hemostasis by increasing the volume and strength of the clot on surgical surfaces. Good et al^[26]^ showed that intravenous TXA administration reduced drain volumes by 50%. Lin et al^[27]^ revealed that blood loss was reduced by approximately 20% in patients who underwent TKA with intravenous TXA. Wong et al^[2]^ found that applying topical TXA directly to the surgical wound in TKA reduced postoperative bleeding by 20% to 25%. Roy et al^[28]^ showed that topical TXA application in TKA reduced the amount coming from the drain by 95% after surgery.

It has been shown that a single dose usually suffices for additional systemic applications after surgery. Long-term repeated applications increase both drug exposure and costs without additional benefit. Recently, Wu et al^[29]^ have shown that only systemic TXA application, starting before surgery and continuing for 3 days after surgery, effectively reduces blood loss in TKA and total hip arthroplasty. This efficacy may not be valid in combined methods because, in our study, we found that additional dose systemic administration after perioperative local and systemic administration did not play an effective role in reducing blood loss. Another recent study performed fibrinolytic phenotyping of patients, investigating whether they benefited from prolonged doses of TXA and demonstrated that longer-term administration of TXA may be effective in patients with insufficient fibrinolysis inhibition.^[30]^

Our study has several limitations. First, our study’s groups contain a small number of patients and surgeries were not performed by a single surgeon and with the same prosthesis design. Also, we did not evaluate early functional scores, so we could not reveal the possible positive contribution of TXA on functional outcomes. Our study is retrospective, but we aimed to minimize the risk of bias by narrowing our inclusion and exclusion criteria. Even though many studies in the literature have compared intravenous and topical methods and reported their results, our comparison of drain clamping durations of study patients to controls makes our study unique. We think that our study contributes to the practice of arthroplasty surgery. In the use of TXA to reduce the amount of bleeding and the need for transfusion in knee arthroplasty, it is seen that a single dose of systemic and local application is compelling enough that clamping time and additional systemic application do not have a beneficial effect.

## 5. Conclusion

TXA is an agent that reduces blood loss in patients undergoing TKA, contributes to maintaining hemodynamic stability, and improves the general condition of patients. Although local and systemic TXA applications do not affect the need for blood transfusion compared to only systemic applications, they significantly protect against Hgb decrease and blood loss. Although the effectiveness of drain clamping time or additional dose systemic applications is not significant in local applications, prospective randomized studies are needed in large patient series where application methods are compared. In this context, we think that the routine use of TXA applications in TKA surgery is beneficial regardless of the method.

## Author contributions

**Conceptualization:** Haluk Çabuk, Ramazan Erden Ertürer.

**Data curation:** Kaya Turan, Tuğrul Ergün.

**Formal analysis:** Osman Görkem Muratoğlu.

**Investigation:** Osman Görkem Muratoğlu.

**Methodology:** Osman Görkem Muratoğlu, Haluk Çabuk.

**Resources:** Tuğrul Ergün.

**Supervision:** Haluk Çabuk, Ramazan Erden Ertürer.

**Writing – original draft:** Kaya Turan.

**Writing – review & editing:** Kaya Turan, Tuğrul Ergün, Ramazan Erden Ertürer.
